# The 2017 Bioinformatics Open Source Conference (BOSC)

**DOI:** 10.12688/f1000research.12929.1

**Published:** 2017-10-19

**Authors:** Nomi L. Harris, Peter J.A. Cock, Brad Chapman, Christopher J. Fields, Karsten Hokamp, Hilmar Lapp, Monica Munoz-Torres, Bastian Greshake Tzovaras, Heather Wiencko

**Affiliations:** 1 Lawrence Berkeley National Laboratory, Berkeley, CA, USA; 2 The James Hutton Institute, Dundee, UK; 3 Bioinformatics Core, Harvard T.H. Chan School of Public Health, Boston, MA, USA; 4 High Performance Computing in Biology Group, Carver Biotechnology Center, University of Illinois Urbana-Champaign, Urbana, IL, USA; 5 Smurfit Institute of Genetics, Trinity College Dublin, Dublin, Ireland; 6 Center for Genomic and Computational Biology, Duke University, Durham, NC, USA; 7 Institute for Cell Biology and Neuroscience, Goethe University of Frankfurt, Frankfurt, Germany; 8 Open Bioinformatics Foundation, Dublin, Ireland

**Keywords:** bioinformatics, open source, open science, open data, community science

## Abstract

The Bioinformatics Open Source Conference (BOSC) is a meeting organized by the Open Bioinformatics Foundation (OBF), a non-profit group dedicated to promoting the practice and philosophy of Open Source software development and Open Science within the biological research community. The 18th annual BOSC ( http://www.open-bio.org/wiki/BOSC_2017) took place in Prague, Czech Republic in July 2017. The conference brought together nearly 250 bioinformatics researchers, developers and users of open source software to interact and share ideas about standards, bioinformatics software development, open and reproducible science, and this year’s theme, open data. As in previous years, the conference was preceded by a two-day collaborative coding event open to the bioinformatics community, called the OBF Codefest.

## Introduction

**Figure 1.**
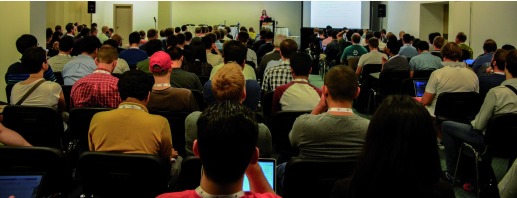
A full house at BOSC 2017 (photo by Bérénice Batut).

The Bioinformatics Open Source Conference, BOSC, has been run every year since 2000 as a two-day Special Interest Group (SIG) before the annual ISMB conference. A record number of nearly 250 people participated in the 18th annual BOSC ( http://www.open-bio.org/wiki/BOSC_2017) ( Figure 1), and around half of these were first-time attendees. The high fraction of first-timers continues a trend observed in recent years, suggesting that the conference’s efforts to increase its outreach to and inclusiveness of new communities is bearing fruit. In connection with these efforts, the OBF in 2016 launched a Travel Fellowship program to help increase the diversity of attendees at open source bioinformatics events, including BOSC; several speakers at BOSC 2017 were recipients of these fellowships.

**Figure 2.**
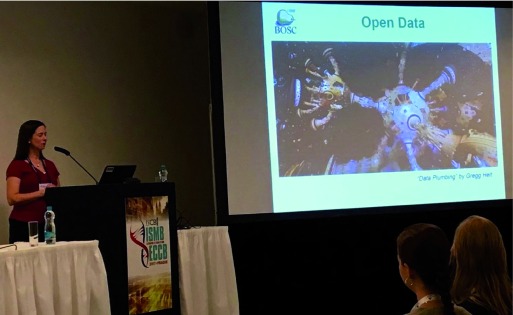
BOSC 2017 chair Nomi Harris introduces the Open Data theme with digital artwork created by Gregg Helt (photo by GigaScience).

BOSC 2017 covered a wide range of topics in open source bioinformatics and open science, including reproducibility; workflows; citizen/participatory science; open source community building; developer tools and libraries for open science; and of course open science and open data. This year's Open Data theme was introduced by chair Nomi Harris ( Figure 2), who cited the FAIR principles of open data: Findability, Accessibility, Interoperability, and Reproducibility ( https://www.force11.org/fairprinciples).

## Keynote speakers

**Figure 3.**
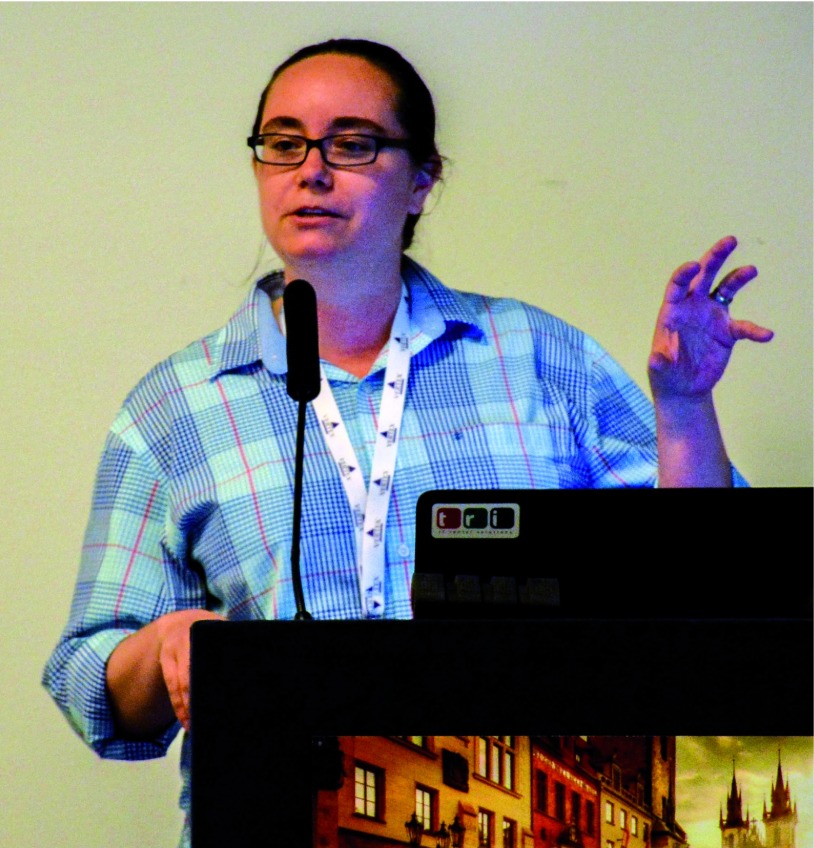
Keynote speaker Madeleine Ball (photo by Nomi Harris).

**Figure 4.**
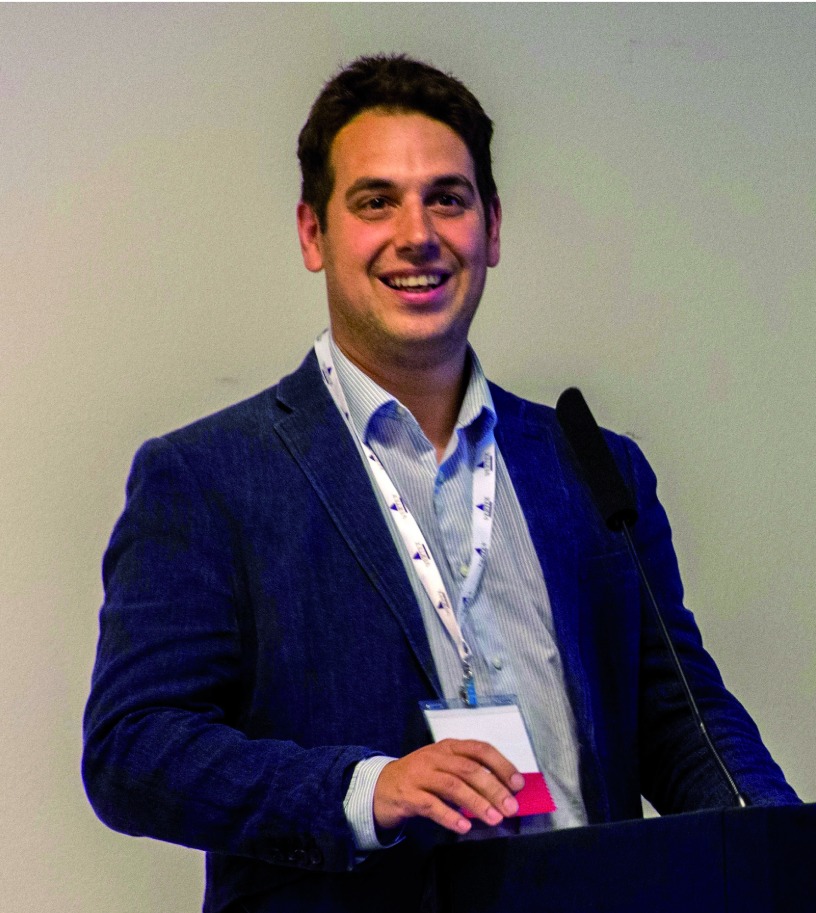
Keynote speaker Nick Loman (photo by Nomi Harris).

Both of this year’s keynote speakers are vocal proponents of open data, particularly in human health research. Madeleine Ball ( Figure 3), the executive director of the Open Humans Foundation ( http://openhumansfoundation.org), spoke about how Open Humans aims to empower people to share their genomes and personal health data with biomedical researchers, while still protecting privacy. The second keynote speaker, Nick Loman of the University of Birmingham ( Figure 4), has been a leader in using open data to expedite effective responses to outbreaks of disease. By combining real-time genomic surveillance of Ebola with open data sharing between research groups, Dr. Loman and colleagues were able to quickly determine where the Ebola outbreak in Guinea started and how it spread across borders.

## Panel discussion

**Figure 5.**
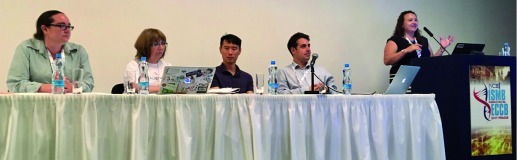
"Open Data: Standards, Opportunities and Challenges" panelists Madeleine Ball, Carole Goble, Andrew Su and Nick Loman, with moderator Monica Munoz-Torres (photo by Nomi Harris).

As in previous years, BOSC included a panel discussion. This year’s topic was “Open Data: Standards, Opportunities and Challenges”. The panelists, who included both keynote speakers as well as Andrew Su (The Scripps Research Institute) and Carole Goble (University of Manchester), engaged audience members in a lively discussion about various issues on both the sharing and the reuse side of open data ( Figure 5). These included misconceptions that are still commonly encountered, such as applying licenses designed for creative works to data, when the real objective is to request compliance with scholarly norms such as proper citation or attribution; and research efficiency-hindering challenges resulting from inadequate scholarly publication venues, such as authors being required to submit supplementary data tables as PDFs, rather than in a reusable format. The panel also touched on some of the confusion surrounding the perception of risks to individuals, endangered species, or the environment, from sharing certain data publicly. For example, responding to the oft-cited worry about publicly shared personal health data potentially threatening one’s ability to obtain health insurance, Madeleine Ball pointed out that at least in some jurisdictions such as the US, patients are already compelled by law to disclose health risks to insurers when asked about them.

## Program

**Figure 6.**
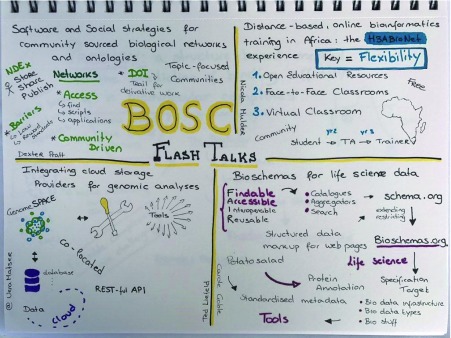
Overview of some of the flash (lightning) talks at BOSC 2017 (sketchnote by Vera Matser).

**Figure 7.**
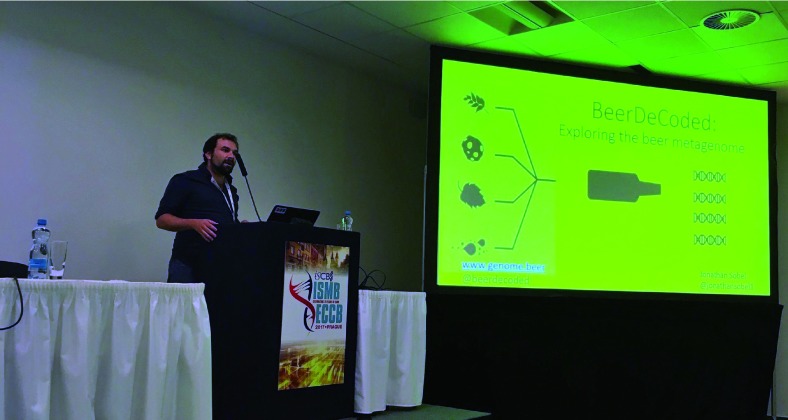
Jonathan Sobel of Hackuarium talking about BeerDeCoded at BOSC 2017 (photo by Bastian Greshake Tzovaras).

To give as many people as possible a chance to present their work, BOSC 2017 included a large assortment of 5-minute lightning talks (see Figure 6 for some highlights), along with a few longer talks, plus over 50 posters. Talk topics included reproducibility, workflows, citizen/participatory science, open source community building, developer tools and libraries for open science, and of course open science and open data. Two subjects, the Common Workflow Language (an open source standard first launched at BOSC 2014), and the Global Alliance for Genomics and Health (GA4GH), attracted enough submissions to comprise their own “groups” within sessions. The popular Community Building and Citizen Science session kicked off with a well-received talk by Jonathan Sobel (one of the OBF Travel award recipients) about the crowdfunded BeerDeCoded project ( Figure 7), which enlisted a non-scientist community to help decode the DNA fingerprint of hundreds of beers, generating a “tree of beers.”

Birds of a Feather sessions—always a great opportunity for BOSC attendees to meet in smaller groups and discuss shared interests—included one on promoting gender diversity in bioinformatics, one about the new Journal of Open Source Software, a CWL discussion, and a conversation about how scholarly publishing should accommodate sharing reproducible research.

## Codefest 2017

The 8th annual collaborative pre-BOSC Codefest ( https://www.open-bio.org/wiki/Codefest_2017) was held July 20–21, 2017, at Brmlab, a community-run hackerspace in Prague. Over 60 attendees worked together on improving and extending a range of old and new open source bioinformatics projects, libraries and standards. Some important outcomes of the Codefest (see summary at https://f1000research.com/slides/6-1187) included welcoming new open source contributors into the community, continuing coordination on widely used standards, finishing last-mile development on integration projects and fixing long standing code issues. For example, the collaborative work on MultiQC ( http://multiqc.info/) specifically focused on new contributors, handling 14 new pull requests for improved functionality from community members. The Common Workflow Language (CWL, http://www.commonwl.org/) standard improved with the addition of reproducible provenance handling, incorporation of HPC-friendly Singularity containers ( http://singularity.lbl.gov/) and updated tooling. The development work and community building at Codefest organizes and energizes the Open Bioinformatics Community for year-round collaboration and open source development.

## 2018 Bioinformatics Community Conference

After many years as part of ISMB, BOSC will be partnering in 2018 with the Galaxy Community Conference as an experiment in broadening the BOSC community. We invite anyone who has an interest in open source bioinformatics or open science to join us in Portland, Oregon, June 25–30—see https://gccbosc2018.sched.com/ for more information.

## Consent

All people in the photos are aware of this publication and are happy to be included in it. The photographers all gave explicit consent for their photos to be used in this report.

## Figures and Tables

**Figure 8.**
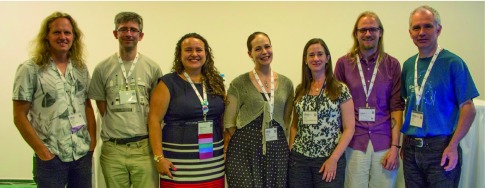
The organizing committee for BOSC 2017: Karsten Hokamp, Peter Cock, Monica Munoz-Torres, Heather Wiencko (co-chair), Nomi Harris (chair), Bastian Greshake and Hilmar Lapp. Not shown: Brad Chapman. (Photo by Jiwen Xin).

